# Isolation of Extracellular Outer Membrane Vesicles (OMVs) from *Escherichia coli* Using EVscore47 Beads

**DOI:** 10.3390/molecules29081831

**Published:** 2024-04-17

**Authors:** Gongming Shi, Xiaohong Yang, Jikai Wang, Wenjing Wei, Kecui Hu, Xingyue Huang, Yanfei Qiu, Yun He

**Affiliations:** 1 Chongqing Key Laboratory of Natural Product Synthesis and Drug Research, School of Pharmaceutical Sciences, Chongqing University, 55 Daxuecheng South Road, Shapingba, Chongqing 401331, China; sgm1234562024@126.com; 2 Chongqing Institute of Green and Intelligent Technology, Chinese Academy of Sciences, 266 Fangzheng Ave, Beibei, Chongqing 400714, China; yangxiaohong@cigit.ac.cn (X.Y.); sinjoy527@163.com (W.W.); hkc03045904@163.com (K.H.); hxy00400314@163.com (X.H.); qyf00009@163.com (Y.Q.); 3 Nanjing Aidimai Technology Co., Ltd., 18 Zhilan Road, Jiangning, Nanjing 211100, China; wangjikaii@163.com

**Keywords:** multifunctional chromatography microspheres, purification, outer membrane vesicles

## Abstract

Outer membrane vesicles (OMVs) are attractive for biomedical applications based on their intrinsic properties in relation to bacteria and vesicles. However, their widespread use is hampered by low yields and purities. In this study, EVscore47 multifunctional chromatography microspheres were synthesized and used to efficiently isolate functional OMVs from *Escherichia coli*. Through this technology, OMV loss can be kept to a minimum, and OMVs can be harvested using EVscore47 at 11-fold higher yields and ~13-fold higher purity than those achieved by means of ultracentrifugation. Based on the results presented here, we propose a novel EVscore47-based isolation of OMVs that is fast and scalable.

## 1. Introduction

Outer membrane vesicles (OMVs) are secreted by various Gram-positive and Gram-negative bacterial strains. They are small, spherical vesicles with a bio-layered structure, typically 10–300 nanometers in size [1]. OMVs contain a variety of biomolecules, including lipids, proteins, and genetic material, which play critical roles in bacteria–bacteria and bacteria–host interactions [2]. Additionally, OMVs are involved in the modulation of the host immune response and can contribute to the pathogenicity of bacteria by delivering virulence factors to host cells [3]. Thus, bacterial OMVs are widely regarded as promising candidates for vaccines [4], adjuvants [5], drug delivery [6], gene transport [7], and anti-bacterial adhesion agents [8]. Currently, several OMV-based vaccines are registered and already on the market. The licensed vaccines such as VA-MENGOC-BC^®^ [9], Men BVac^®^ [10], MeNZB^®^ [11], and Bexsero^®^ [12] are samples of OMV-based vaccines against *Neisseria meningitidis* serogroup B. PedvaxHib (PRP-OMPC) [13] is licensed widely and targets *Haemophilus influenzae* type b (Hib) infection. Another two OMV-based vaccines, Avacc 10 against COVID-19 and iNTS-GMMA against invasive non-typhoidal *Salmonella*, are already in Phase 1; a third, altSonflex1-2-3 against *Shigella*, is Phase 2 [4]. In addition, OMV-based vaccines for anti-cancer are also being studied by several groups [14,15,16]. OMVs have diverse functions and many bio-applications, and high-yield and high-purity OMVs are a prerequisite to developed good OMV-based vaccines or drugs.

Currently, OMVs can be purified using various techniques, such as ultracentrifugation [17], ultrafiltration [18], protein precipitation [19], affinity isolation [20], and size-exclusion chromatography [21]. Ultracentrifugation remains the most common method used to isolate bacterial OMVs [22]. This technique typically involves multiple rounds of centrifugation at progressively higher speeds to effectively harvest and concentrate the OMVs. However, this process is time-consuming. Additionally, centrifugal force can lead to the destruction of the exosome structure; this, in turn, affects downstream experiments, particularly in the functional analysis of exosomes [1]. Ultrafiltration is another preferred method for extracting OMVs. However, membrane clogging can be a common problem when using ultrafiltration [23]. For protein precipitation methods, OMVs are separated via the addition of high concentrations of salts such as ammonium sulfate (AS). In this process, the high salt concentrations destroy the balance of surface charges and hydrogen bonds of proteins, making the proteins insoluble. The yield of OMVs obtained via AS precipitation may be enhanced compared to that obtained via ultracentrifugation or ultrafiltration, but the purity of OMVs isolated using this technique is generally low [22,23]. Affinity isolation has been applied to the rapid purification of OMVs. The immobilized metal affinity chromatography (IMAC) Ni-NTA resin was applied to purify OMVs of genetically engineered bacteria with a non-native histidine amino acid repeat sequence (His-tag) [20]. This technology can directly purify OMVs from bacterial media, but it requires the addition of the His-tag to a membrane protein of the isolated bacterium, which is limited to the vesicles from common bacteria. Size-exclusion chromatography can purify vesicles because of their large molecular weight compared to other soluble components in the medium. However, the column volume limits the amount of sample that can be processed, and the yields and purity are low [24]. Thus, there is an urgent need for more efficient and reliable OMV isolation protocols, so that the yield and purity can meet industrial and clinical needs.

Recently, Capto Core 700 columns have been developed for the isolation of exosomes from cell cultures and blood plasma [25,26,27]. Capto Core 700 is a core-shell adsorbent resin bead and applied to purify large biomolecules and bioparticles in a flow-through mode. The Capto Core beads differ from Sephadex or other size-exclusion matrices, combining size separation with bind/elute chromatography [28]. For Capto Core 700 beads, smaller molecules (<700 kDa) penetrate the inert outer shell and bind to hydrophobic and positively charged octylamine ligands within the core, while large molecules bypass these beads [25]. Capto Core 700 beads have been also applied to purify large proteins [29], virus [30], and mesenchymal stem cells [31]. However, the literature is limited to the application of OMV separation by Capto Core beads. In fact, Capto Core 700 consists of a single agarose-base matrix and its core and shell have the same porous structure, so the pores of resin beads might be easily blocked by different sizes and various shapes of molecules.

Thus, the main objectives of this study were to synthesize EVscore47 multifunctional chromatography microspheres for the efficient isolation of OMVs from *Escherichia coli* (*E. coli*) and to assess the yield and separation efficiency of the applied protocol. The core and shell of EVscore47 had different porous structures, which were designed to remove different sizes and various shapes of contaminants in the bacteria medium. The mixed-mode microsphere consists of an adsorptive core with hydrophobic and ionic interactions and a hydrophilic shell with an exclusion effect. The core was formed by the bonding of ionizable amino long chain alkane with agarose microsphere resin. The shell was formed by the internal impregnation and external deposition of agarose, glucan, cellulose, and other polysaccharides. The thickness and exclusion limit of the exclusion layer can be controlled by the porosity of the agarose microsphere, the concentration of polysaccharide, and the cross-linking degree of the shell layer. No highly toxic reagents were added into the reaction system, making it safe for downstream purification and especially for the isolation of OMVs. This system combines the functions of volume-exclusion chromatography, ion chromatography and hydrophobic chromatography to achieve rapid purification of OMVs from complex bacterial samples.

## 2. Results and Discussion

### 2.1. Preparation and Characterization of EVscore47 Microspheres

**Figure 1:**
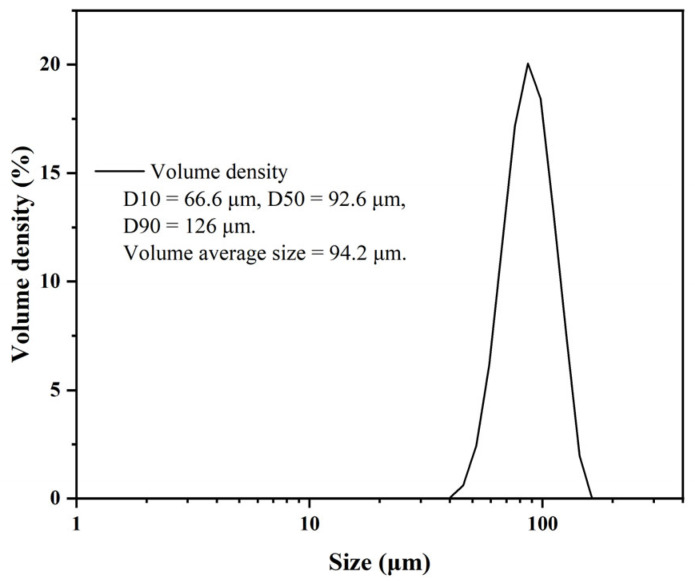
Particle-size distribution of EVscore47 microspheres.

The EVscore47 multifunctional chromatography microspheres were synthesized via three steps: (a) crosslinking and activation of agarose microspheres, (b) ionizable hydrophobic molecular bonding, and (c) coating of the shell layer. Figure 1 shows the particle-size distribution (PSD) of the EVscore47 microspheres. The particle diameter of the beads was 66–126 μm, and their volume-average diameter was 94.2 μm.

**Figure 2:**
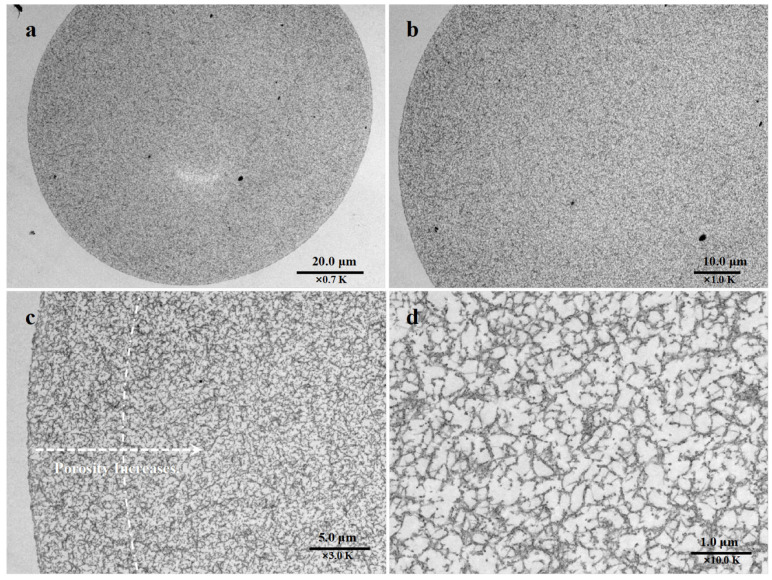
TEM images of EVscore47 resin at different magnifications. (**a**) a section of bead at 0.7K magnification; (**b**) a section of bead at 1.0K magnification; (**c**) a section of bead at 3.0K magnification; (**d**) a section in the core area of bead at 10.0K magnification.

The morphology of the EVscore47 microspheres was characterized via transmission electron microscopy (TEM). The resin backbone was stained by the heavy-metal mixture and found in the darker regions of the TEM images. As shown in Figure 2a, the beads showed a fibrous architecture and appeared to have the same backbone structure across the radius. However, the shell and core regions displayed different backbone-structure densities (Figure 2b). The backbone density near the surface area was higher than that in the core area, as the surface area was darker due to the high backbone density. Furthermore, this difference was more obvious at higher magnifications (×3.0 K). As can be seen from Figure 2c, the porosity increased in the direction of the auxiliary arrow, and the dashed arc approximately divides the high-density shell and low-density core areas. The porosity of the outer shell area was approximately one-third of that of the core area. As desired, the smaller porosity could be expected to filter out relatively few molecules. Figure 2d shows the higher-resolution image of the core area. At this magnification, larger pores were observed, which could be expected to adsorb more molecules.

**Figure 3:**
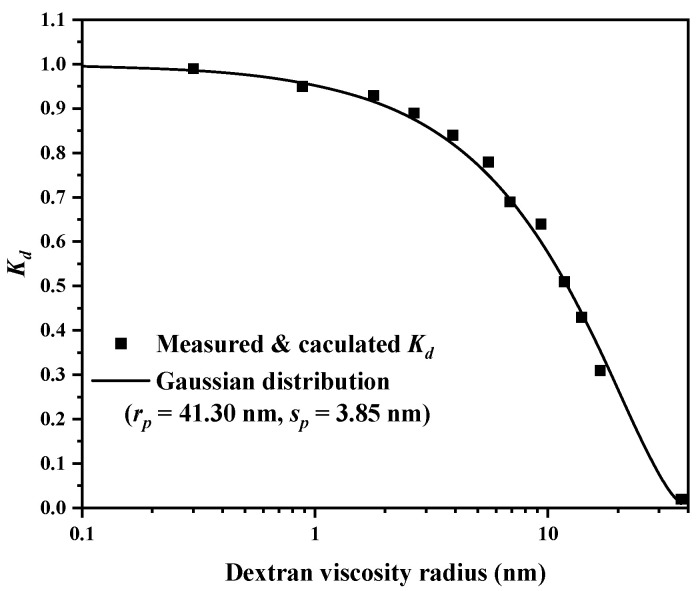
The *K_d_-R_m_* relationship and the Gaussian fitted curve (*K_d_*, the theoretical distribution coefficient; *R_m_*, dextran-viscosity radius; *r_p_*, the mean of the distribution; *s_p_*, the standard deviation of the distribution).

**Figure 4:**
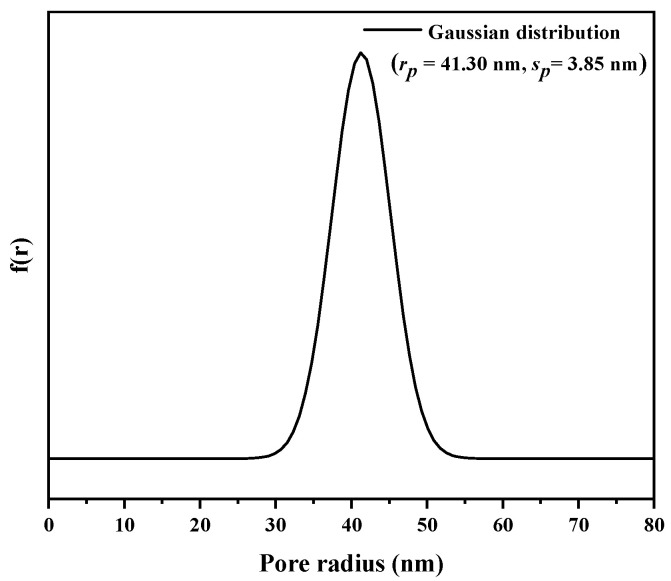
Pore-radius distribution of EVscore47 calculated with the Gaussian model.

Inverse size-exclusion chromatography (iSEC) was applied to measure the pore size of the EVscore47 microspheres. The main parameters of the dextran and deoxyribonucleic acid (DNA) standards are listed in Table S1. The relationship between *K_d_* and the viscosity radius (*R_m_*) was fitted with a Gaussian distribution, and the fitted curve is shown in Figure 3. The measured data were consistent with the fitted Gaussian distribution curve, and the calculated *r_p_* and *s_p_* values were 41.30 nm and 3.85 nm, respectively. The pore-radius distribution with the Gaussian model is shown in Figure 4.

### 2.2. Adsorption Isotherms

**Figure 5:**
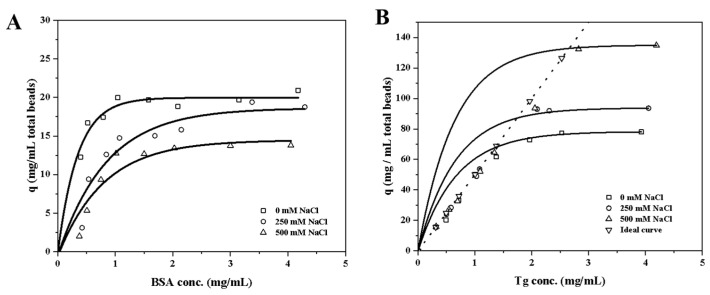
Binding capacities for (**A**) BSA and (**B**) Tg, determined after 24 h of incubation in 20 mM Tris-HCl at pH 7.5 with 0, 250, and 500 mM of NaCl. The ideal curve indicates the theoretical capacities of Tg at the definite concentration of protein. BSA, bovine serum albumin; Tg, thyroglobulin.

Both bovine serum albumin (BSA) and thyroglobulin (Tg) proteins were expected to bind to EVscore47 at a neutral pH. The molecular weights of BSA and Tg are 66 kDa and 660 kDa, respectively, while the hydrodynamic radius is 3.7 nm for BSA and 8.7 nm for Tg. The Langmuir isotherm model was applied to analyze the protein-adsorption behavior: *q* = *q_m_K_L_*C/(1 + *K_L_*C), where *q_m_* is the maximum binding capacity (mg/mL of total bead volume) and *K_L_* is the Langmuir affinity constant (mL/mg of protein). The fitted parameters are shown in Table 1, and the adsorption isotherms are shown in Figure 5. The EVscore47 resins displayed high binding capacities for both BSA and Tg, and the binding capacity increased with increasing protein size. As can be seen from Figure 5A, the binding strength decreased as the salt concentration increased for the BSA-resin system. However, the variation tendency was the opposite for Tg at 250 mM and 500 mM Na^+^ (Figure 5B). To further compare the adsorption behavior of BSA and Tg at the same concentration of protein by weight, the theoretical ideal curve was established to explain the adsorption behavior of EVscore47 at low Tg contents. In Figure 5B, the points marked under the curve indicate the real adsorption behavior, while the dots on the ideal curve mean that all Tg molecules were adsorbed onto the EVscore47 resins under those conditions (Figure 5B). This difference resulted from the combined effects of electrostatic interaction, hydrophobic adsorption, and molecular exclusion. Electrostatic interaction wanes when salt is added, and the binding becomes dominated by hydrophobic forces. A high salt content enhances the hydrophobic adsorption in the confined space of the pore path, enabling greater adsorption of the larger protein Tg.

### 2.3. Confocal Laser Scanning Microscopy (CLSM) Results

**Figure 6:**
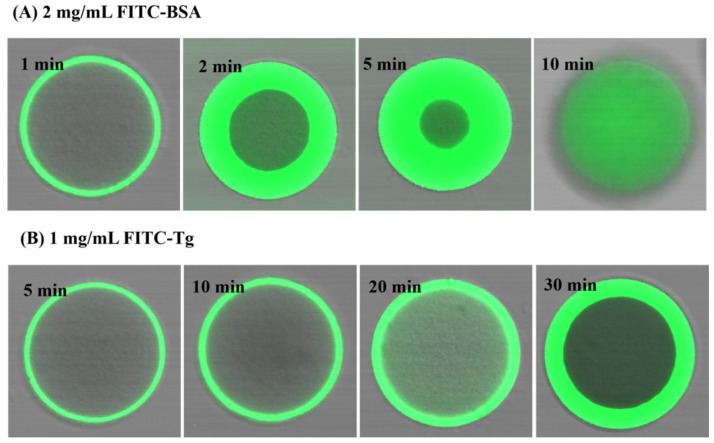
CLSM images of EVscore47 particles during adsorption of BSA and Tg in 20 mM Tris-HCl at pH 7.5: (**A**) 2 mg/mL FITC-labeled BSA, (**B**) 1 mg/mL FITC-labeled Tg in 500 mM NaCl. The times are shown in each image. The particles shown in the images have diameters of 110 ± 10 μm.

To further investigate the transient adsorption of the proteins BSA and Tg on EVscore47 resin, the protein–resin system was observed via confocal laser scanning microscopy (CLSM). The fluorescein isothiocyanate (FITC)-labeled BSA or Tg was incubated gently with EVscore47, and then the protein adsorption could be visualized via CLSM. Figure 6 shows representative CLSM images for the adsorption kinetics of 2 mg/mL FITC-labeled BSA without NaCl and 1 mg/mL FITC-labeled Tg with 500 mM NaCl on the EVscore47 spheres. The FITC protein penetrated into the EVscore47 spheres, where it appeared green under CLSM. As shown in Figure 6A, BSA binding occurred rapidly on the EVscore47 spheres, which were nearly completely saturated with BSA in just 10 min. However, the Tg adsorption kinetics were much slower, as indicated by the nearly uniform saturation of the bead at 30 min (Figure 6B). The difference in permeation velocity might have been due to the molecular size difference between BSA (66 kDa) and Tg (660 kDa). The low permeation velocity of the Tg protein indicates that the size of the Tg protein is close to the exclusion limit of EVscore47 shells. Additionally, short- and long-term binding of BSA and Tg essentially occurs only in the core.

### 2.4. Isolation and Characterization of OMVs

**Figure 7:**
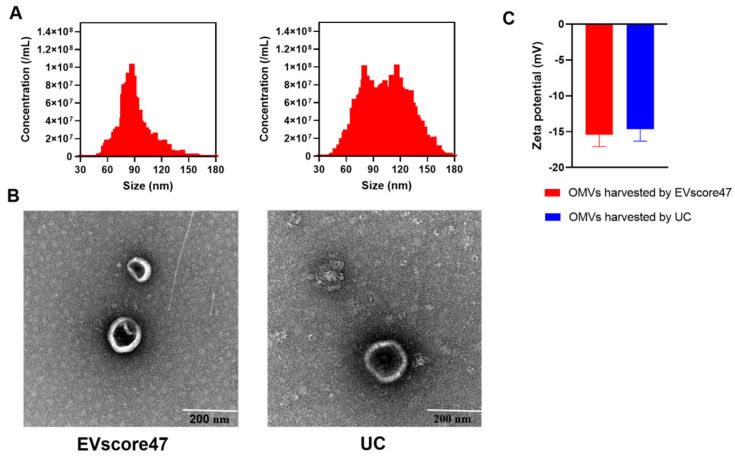
Morphological analysis of outer membrane vesicles (OMVs) isolated with EVscore47 and ultracentrifugation (UC): (**A**) size distribution of OMVs (triplicate); (**B**) TEM images of OMVs; (**C**) zeta potential of OMVs.

OMVs from *E. coli* were isolated by means of EVscore47 and ultracentrifugation (UC), respectively. The size distribution and zeta potential of the OMVs were measured using NanoFCM. As shown in Figure 7A, the diameter of the OMVs purified using EVscore47 (75.8 nm) was slightly smaller than that of the OMVs isolated by means of UC (85.4 nm). The zeta potential (Figure 7C) of the OMVs harvested using EVscore47 (−15.41 ± 1.66 mV) was similar to that of the OMVs harvested by means of UC (−14.68 ± 1.62 mV). To verify the OMVs’ sizes and morphology, individual OMVs were visualized by means of negative staining transmission electron microscopy (TEM). The TEM images showed that the OMVs isolated using EVscore47 had a bilayer structure and a round morphology, with diameters ranging from 40 to 200 nm, similar to those obtained by means of UC (Figure 7B). These data indicate that the OMVs could be successfully purified using the EVscore47 resin. UC is the traditional, standard method to obtain OMVs. However, the UC method requires multi-step progressive centrifugation. In fact, some destruction of vesicles obtained by means of UC was observed under TEM, which might have been due to the high centrifugal force. This phenomenon was not found in the OMVs isolated using EVscore47. In our experiments, for 100 mL of bacterial medium, the isolation with the beads only required 1.5 h, while it took 7 h to obtain OMVs via the UC technique. The purification with EVscore47 beads does not require multi-step centrifugation and ultrahigh-speed centrifugation, so the separation progress is faster.

### 2.5. Amounts of OMVs

**Figure 8:**
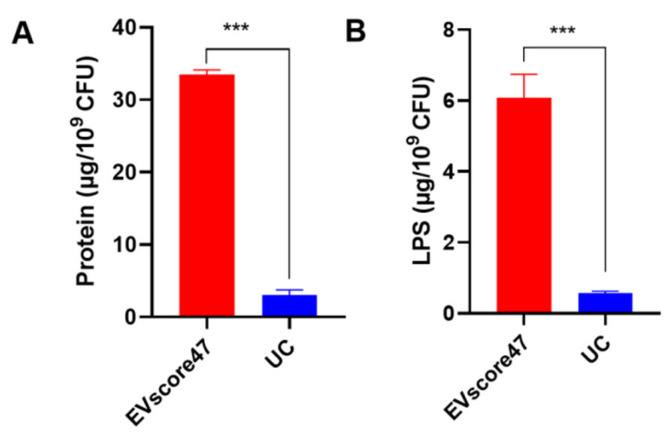
Comparison of OMV yields harvested using EVscore47 and ultracentrifugation (UC): (**A**) The total amounts of protein in the OMVs were measured by means of a bicinchoninic acid protein assay. (**B**) The lipopolysaccharide (LPS) contents of OMVs were determined via enzyme-linked immunosorbent assay. Harvesting yields were normalized with respect to the number of *E. coli* CFUs involved; *** *p* < 0.001.

The total amounts of protein and lipopolysaccharide (LPS) were used to compare the amounts of OMVs separated by means of EVscore47 and UC. UC is the traditional, standard method to obtain OMVs, but the isolation process is very complex and the yield is extremely low. As shown in Figure 8, the total amounts of protein and LPS in OMVs separated using EVscore47 were almost 11-fold higher than in those obtained via UC. These data indicate that the isolation with EVscore47 beads resulted in increased yields of OMVs. This purification method does not require cumbersome centrifugation, nor does it require the addition of any His-tag to the bacteria or chemicals to the bacterial medium, compared to the reported affinity isolation [20] and protein precipitation methods [19]. The separation process becomes simpler and safer, which might reduce losses during extraction and contribute to the higher quantities of harvested OMVs.

### 2.6. Protein Composition of OMVs

**Figure 9:**
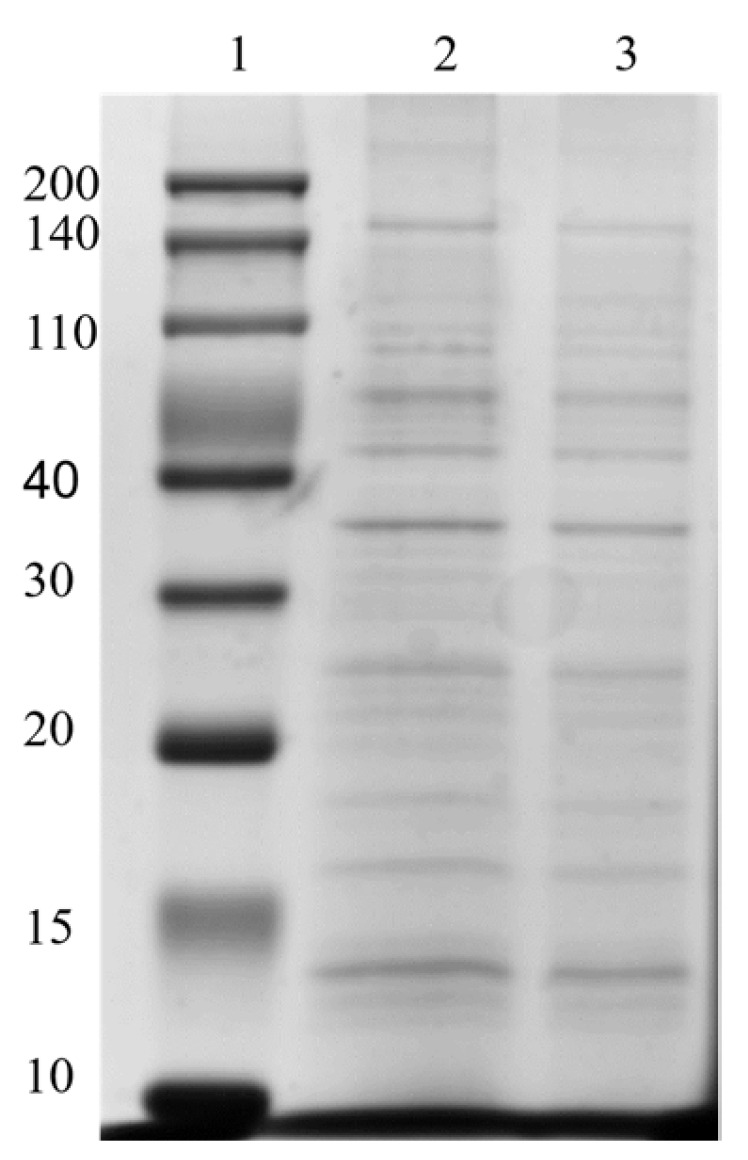
Protein analyses of OMVs harvested using SDS-PAGE. Gels were stained with Coomassie blue. 1, protein markers; 2, OMVs harvested using EVscore47; 3, OMVs harvested by means of UC.

To determine the protein composition of OMVs prepared from *E. coli*, 20 µg of protein was subjected to 12% SDS-PAGE. As shown in Figure 9, approximately 10 major protein bands, ranging in molecular weight (MW) from 10 to 200 kDa, were identified in *E. coli* OMVs purified using EVscore47. The estimated MWs of these protein bands were ~15, ~16, ~18, ~25, ~38, 40–110, and 140–200 kDa. The OMV proteins (EVscore47) that formed thick bands in the gel were highly similar to those of the OMVs obtained via ultracentrifugation, suggesting that the OMVs isolated via the two methods had similar protein profiles. These data further demonstrate that EVscore47 successfully purified OMVs from the bacterial medium.

### 2.7. Purity of OMVs

**Figure 10:**
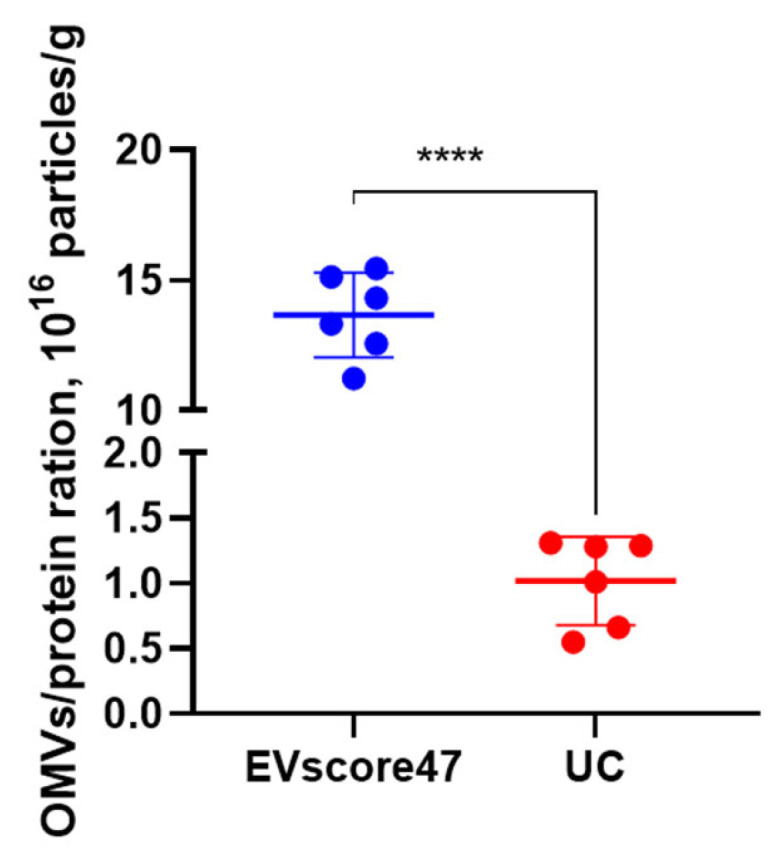
Analysis of the particle–protein ratio in the exosomes isolated from *E. coli* using two different isolation methods; **** *p* < 0.0001.

To determine the OMVs’ purity, the ratio between the particle concentration and protein content was calculated for each of the two methods. The particle concentration and protein content were measured using NanoFCM and the BCA protein assay kit, respectively. As shown in Figure 10, higher ratios (13.33 × 10^16^ P/g protein) were achieved with EVscore47, while UC achieved lower ratios (0.63 × 10^16^ P/g protein). EVscore47 increased the OMVs’ purity by ~13-fold compared to the UC method. Combining the data mentioned above, the EVscore47 separation harvested high-yield OMVs, but the increased yield did not decrease the purity of the OMVs, suggesting that the EVscore47 beads could be applied in industrial purification.

## 3. Materials and Methods

### 3.1. Materials

*E. coli* ATCC25922 was purchased from Beina Biotechnology Co., Ltd. (Beijing, China). Luria–Bertani (LB) medium was obtained from Qingdao Haibo Biotechnology Co., Ltd. (Qingdao, China). Phosphate-buffered saline (PBS, pH = 7.4) was purchased from Wuhan Saiweier Biotechnology Co., Ltd. (Wuhan, China). The bicinchoninic acid (BCA) protein assay kit and bovine serum albumin (BSA) were purchased from Sango Biotech (Shanghai, China). The thyroglobulin (Tg) from bovine thyroid was obtained from Shanghai Yuanye Biological Technology Co. (Shanghai, China). Ultrapure water (Millipore Milli-Q grade, 18.2 MΩ) was used in all of the experiments. All chemicals were used as received, without special purification, unless stated otherwise.

### 3.2. Preparation of EVscore47 Microspheres

The EVscore47 multifunctional chromatography microspheres were synthesized as follows. Briefly, 400 mg of dry 4% agarose resin powder was dissolved in 20 mL of 1 M sodium hydroxide solution containing 0.5% sodium borohydride. Then, 2 mL of epichlorohydrin was added to the solution and kept at 37 °C for 2.5 h. Once cooled, the solution was washed thrice with deionized water to obtain the crosslinked spheres. The activation process was similar to the crosslinking steps, while the dosage of epichlorohydrin was doubled to ensure a high epoxy density.

Next, the aforementioned activated agarose resin microspheres were dispersed in 150 mL of 0.1 M sodium hydroxide solution. Then, 200 mL of n-hexylamine was added to the solution and stirred at 37 °C for 24 h. Finally, the product was washed thrice with deionized water to obtain the n-hexylamine-grafted agarose resin microspheres.

Finally, the n-hexylamine-grafted agarose resin microspheres were dispersed in 100 mL of deionized water and stirred at 60 °C; 0.4 g of dextran and 0.4 g of agar were dissolved in 10 mL of deionized water, which was poured to the microsphere mixture to dip and coat the shells. Next, the coated agarose resin microspheres were separated by size and washed through the filter with deionized water to obtain the EVscore47 spheres.

### 3.3. Structural Properties

The particle-size distribution (PSD) of EVscore47 was measured using the Malvern Mastersizer3000 (Malvern Instruments, Malvern, UK). Transmission electron microscopy (TEM) was used to observe the intraparticle nanoscale structure of EVscore47. The resin samples were dehydrated by washing them with increasing ethanol concentrations, from 0 to 100%. Each sample was then saturated with LR White resin (Macklin, Shanghai, China) and ultramicrotomed into 80 nm sections using a Leica MZ6 Ultracut ultramicrotome (Leica ultracut, UCT, England), and sections of the resin samples were stained with a mixture of uranyl acetate and lead citrate. Finally, these sections were imaged with an 80 kv TEM (JEOL JEM-2100Plus, JEOL Ltd., Tokyo, Japan) at 0.7, 1.0, 3.0, and 10k magnification, as described in [28].

The pore-size distribution of EVscore47 was measured via inverse size-exclusion chromatography (iSEC) based on different dextran and DNA standards, as described previously [32,33]. The most frequently used local partition coefficient model was adopted, with the assumption of rigid spherical solutes in the cylindrical pores, and then the relationship between PSD and the theoretical distribution coefficient (*K_d_*) was established.

The value of *K_d_* could be measured and calculated using the following relation:

(1) $$K_{d}=\frac{V_{R}-V_{0}}{V_{T}-V_{0}}$$

where *V_R_* is the solute elution volume, *V*_0_ is the interparticle void volume, and *V_T_* is the total volume of the mobile phase.

Based on the Model (2) assumption of rigid spherical molecules into the columnar pores and Gaussian Relation (3),

(2) $$K={\left(1-\frac{R_{m}}{r}\right)}^{2}$$

(3) $$f(r)=\exp[-\frac{1}{2}{\left(\frac{r-r_{p}}{s_{p}}\right)}^{2}]$$

where *r* is the pore size, *R_m_* is the pore size of the probe molecule, and *r_p_* and *s_p_* are the mean and standard deviation of the distribution, respectively.

The relationship between the PSD and *K_d_* can be established as follows:

(4) $$K_{d}=\frac{\int_{R_{m}}^{\infty }f(r)[1-{\left(R_{m}/r\right)}^{2}]dr}{\int_{0}^{\infty }f(r)dr}=\frac{\int_{R_{m}}^{\infty }e^{-\frac{1}{2}{\left(\frac{r-r_{p}}{s_{p}}\right)}^{2}{\left(1-\frac{R_{m}}{r}\right)}^{2}dr}}{\int_{0}^{\infty }e^{-\frac{1}{2}{\left(\frac{r-r_{p}}{s_{p}}\right)}^{2}dr}}$$

### 3.4. Adsorption Isotherms

Adsorption capacities were measured in 20 mM Tris-HCl buffer with 0, 250, or 500 mM NaCl at pH 7.5, as described previously [28]. Briefly, each resin sample was incubated with different concentrations of BSA or Tg solution and rotated end-over-end for 24 h. Then, the residual protein concentration in the solution was measured using a NanoDrop 2000 UV–Vis spectrophotometer (Thermo Scientific, Wilmington, DE, USA), and the adsorbed protein concentration was calculated from the mass balance. The hydrated resin densities were measured with a pycnometer and found to be 1.06 g/mL of hydrated particle for the resins, which was used to convert the adsorbed concentrations to units of mg per mL of hydrated bead volume.

### 3.5. Confocal Microscopy

Confocal laser scanning microscopy (CLSM) was used to image the transient adsorption of the BSA and Tg proteins on EVscore47 resin, and the measurements were performed with fluorescently labeled tracers as described previously, with minor revisions [28]. To observe the adsorption behavior of different-sized proteins, both BSA (66 kDa) and Tg 660 (kDa) were selected as model proteins. The BSA and Tg proteins were labeled with fluorescein isothiocyanate (FITC). Each labeled protein was diluted with a sufficient quantity of unlabeled protein. The labeled-to-unlabeled protein ratios used in these experiments were 1:50 and 1:40 for BSA and Tg, respectively. At different times following addition of the resin, imaging was performed using a Zeiss 510 confocal microscope (Carl Zeiss, Oberkochen, Germany).

### 3.6. Isolation and Characterization of OMVs from E. coli

#### 3.6.1. Bacterial Culture

*E. coli* ATCC25922 strains were used in this study. The *E. coli* strains were cultured on LB agar plates. For pre-culturing, one colony of each strain was transferred to 5 mL of LB medium and cultured at 37 °C overnight. Then, 2 mL of this preculture was added to fresh LB medium (200 mL) and grown for another 24 h at 37 °C.

#### 3.6.2. Sample Processing

The bacterial culture suspensions were centrifuged at 5000× *g* for 10 min at 4 °C to pellet the bacteria. Subsequently, the supernatants were filtered through a 0.22 µm filter (Pall corporation, Port Washington, NY, USA) to ensure the removal of all bacteria. The filtrate was used to purify the OMVs in the next step.

#### 3.6.3. Purification of OMVs Using EVscore47 Beads

The EVscore47 columns (5 mL) were equilibrated with 5 mL of ultrapure water, and the prepared bacterial medium described above was added to the EVscore47 column. Fractions with OMVs were collected into sterile centrifuge tubes. The EVscore47 column was regenerated with 1 M NaOH in 30% 2-propanol solution.

#### 3.6.4. Isolation of OMVs via Ultracentrifugation

The prepared bacterial medium described above was centrifuged at 100,000× *g* (Beckman Corp, Brea, CA, USA) for 3 h at 4 °C. Then, the pellet was washed in PBS and centrifuged at 120,000× *g* for 3 h. Finally, the OMVs were resuspended in PBS and stored at −80 °C prior to use.

#### 3.6.5. Characterization of OMVs

The particle-size distribution and zeta potential of the OMVs were measured in PBS at 25 °C using the NanoFCM N30E Flow NanoAnalyzer (NanoFCM Co., Ltd., Nottingham, UK). For transmission electron microscopy (TEM), 20 µL OMV samples were adsorbed onto a carbon-coated copper grid and allowed to attach for 30 min. Then, the grids were negatively stained with 2% phosphotungstic acid hydrate. The morphology of the OMVs was observed under TEM at 100 kV (JEOL JEM-2100Plus, JEOL Ltd., Tokyo, Japan).

#### 3.6.6. Protein and Lipopolysaccharide Contents in OMVs

The yields of OMVs harvested from bacteria using EVscore47 and ultracentrifugation were compared based on their protein and lipopolysaccharide contents. For the determination of protein contents, the OMVs were lysed using RIPA buffer (20 min on ice). Then, the resulting mixture was centrifuged at 15,000× *g* for 10 min at 4 °C, and the supernatant was used to determine the protein content. The protein content was measured using a bicinchoninic acid (BCA) protein assay kit (Beyotime, Shanghai, China), and the UV–vis absorbance was recorded at 562 nm using a SpectraMax^®^ i3x microplate reader (Molecular Device, Jose, CA, USA). A standard curve using serial dilutions of a BSA solution was used to determine the total protein concentration of the samples. All samples and standards were applied to the well plates in triplicate.

Lipopolysaccharide (LPS) is an universal marker for the Gram-negative bacterial OMVs, with well-characterized, commercially available affinity reagents [34]. In many species, including *E. coli*, specific markers of extracellular vesicles (EVs) and non-EV materials remain unavailable [35]. Therefore, it is difficult to identify OMVs by Western blot, especially for the non-genetically engineered bacteria. Because OMVs contain significant quantities of LPS, the LPS content of OMVs was determined by ELISA in the previous report [36]. In our present study, the LPS content was measured using an enzymelinked immunosorbent assay (ELISA) kit (Shanghai Enzyme-linked Biotechnology Co., Ltd., Shanghai, China). According to the manufacturer’s instructions, the lysed OMVs were added to wells containing LPS antibody, mixed with 100 μL of HRP-conjugated reagent, and incubated for 60 min at 37 °C. Subsequently, the plates were washed five times with a wash solution, and 3,3,5,5-tetramethylbenzidine (TMB) was added to visualize bound IgG. The optical density (O.D.) at 450 nm was read using a microtiter plate reader (Molecular Device, San Jose, CA, USA) within 15 min.

#### 3.6.7. Protein Composition of OMVs

Sodium dodecyl sulfate–polyacrylamide gel electrophoresis (SDS-PAGE) was used to analyze the protein composition of the OMVs. The OMVs were mixed with SDS-PAGE sample buffer (Beyotime, Jiangsu, China) and immersed at 90 °C for 10 min. After that, the mixture was centrifuged at 12,000 rpm for 10 min at 4 °C, and the supernatant was used for SDS-PAGE. Then, 20 µg samples were placed on a 12% (wt/vol) polyacrylamide gel. Finally, the gel was stained with Coomassie blue and imaged using a ChemiDoc system (Bio-Rad, Shanghai, China). Standard protein markers (Beyotime, Jiangsu, China) were used as molecular weight markers, ranging from 10 to 200 kDa.

### 3.7. Statistical Analysis

Data are expressed herein as the mean ± standard deviation (SD). Differences between groups were analyzed using Student’s *t*-test or one-way ANOVA. GraphPad Prism (9.5.1) was used for statistical analyses. Values of *p* < 0.05 were considered statistically significant; *p*-values are indicated as * for *p* < 0.05, ** for *p* < 0.01, *** for *p* < 0.001, and **** for *p* < 0.0001.

## 4. Conclusions

In the present study, EVscore47 multifunctional chromatography microspheres were synthesized and successfully applied to purify OMVs from an *E. coli* medium. OMVs could be harvested using EVscore47 at 11-fold higher yields than those achieved by means of ultracentrifugation. Moreover, the purity of the OMVs obtained using EVscore47 increased by ~13-fold compared to that of OMVs obtained using the UC method. Additionally, EVscore47 beads make the isolation process simple, reducing the separation time from 7 h (UC method) to 1.5 h. Meanwhile, the protein composition of the OMVs obtained using the beads was similar to that of those obtained via the gold-standard UC method. Based on these data, we propose that EVscore47 beads could be used for the industrial purification of OMVs or exosomes. Further studies should explore the possibility of using EVscore47 beads for purification in other kinds of bacteria, cells, or plants. This study provides a new method for isolating OMVs.

## Figures and Tables

**Table 1:** The fitted *q_m_* and *K_L_* values of the Langmuir model.

Protein	0 mM NaCl		250 mM NaCl		500 mM NaCl	
	*q_m_*	*K_L_*	*q_m_*	*K_L_*	*q_m_*	*K_L_*
BSA	20.45	9.54	20.13	1.81	15.58	1.94
Tg	84.04	2.61	100.15	2.77	143.44	2.92

Note: *q_m_* is the maximum binding capacity (mg/mL of total bead volume); *K_L_* is the Langmuir affinity constant (mL/mg).

## Data Availability

All data are contained within the article.

## Supplementary Materials

The following supporting information can be downloaded at: https://www.mdpi.com/article/10.3390/molecules29081831/s1, Table S1: Main parameters of dextran standards and deoxyribonucleic acid (DNA) standard.
