# Supplementary material for: Isolation of Extracellular Outer Membrane Vesicles (OMVs) from Escherichia coli Using EVscore47 Beads
Source: Molecules. 2024 Apr 17;29(8):1831. doi: 10.3390/molecules29081831 (PMC11054804; doi:10.3390/molecules29081831)
Supplement: Supplementary file 1 [file molecules-29-01831-s001.zip › molecules-2931561-supplementary.pdf]

Table S1. Main parameters of dextran standards and deoxyribonucleic acid (DNA) standard.

Name	Mp	Mw	Mn	PDI	R_m/nm
dextran	1080	1270	1010	1.26	0.88
dextran	4440	5220	3260	1.60	1.78
dextran	9890	11600	8110	1.43	2.65
dextran	21400	23800	18300	1.30	3.89
dextran	43500	48600	35600	1.36	5.53
dextran	66700	80900	55500	1.46	6.85
dextran	123600	147600	100300	1.47	9.31
dextran	196300	273000	164200	1.66	11.72
dextran	276500	409800	236300	1.73	13.90
dextran	401300	667800	332800	2.01	16.73
dextran	2000000				37.23
calf thymus DNA		8600000			

Note: Mp, peak molecular mass; Mw, weight-average molecular weight; Mn, number-average molecular weight; PDI, the polydispersity; R_m, dextran viscosity radius.
